# Supplementary material for: Transcriptomic Analysis on the Effects of Altered Water Temperature Regime on the Fish Ovarian Development of Coreius guichenoti under the Impact of River Damming
Source: Biology (Basel). 2022 Dec 15;11(12):1829. doi: 10.3390/biology11121829 (PMC9775624; doi:10.3390/biology11121829)
Supplement: Supplementary file 1 [file biology-11-01829-s001.zip › biology-2030802-supplementary.pdf]

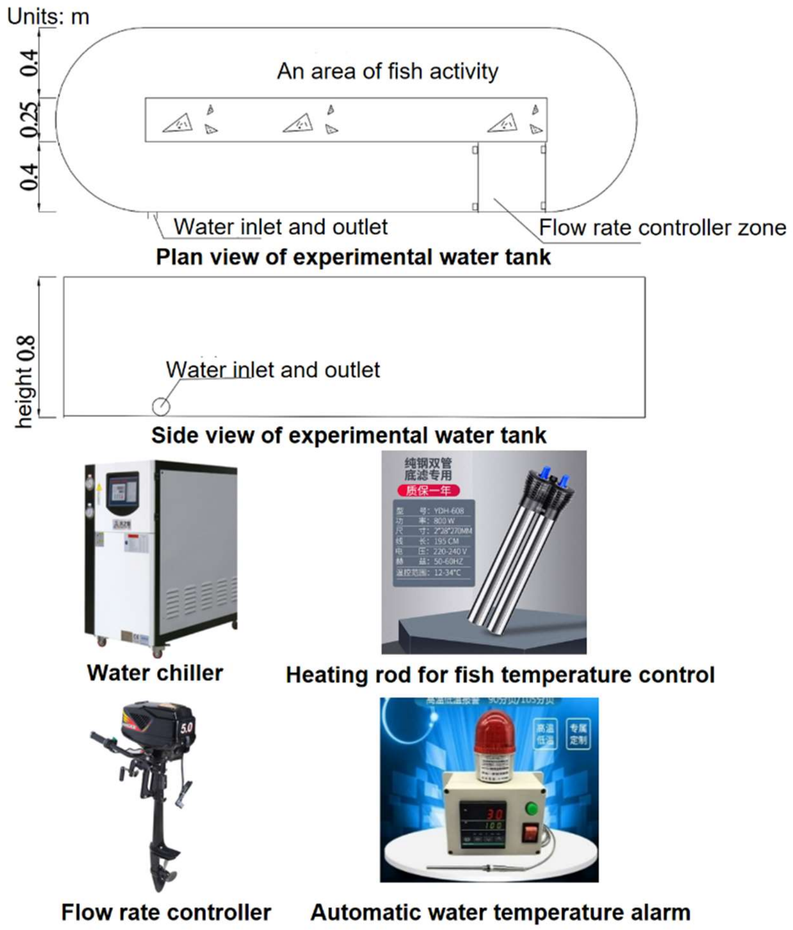

Figure S1 Experimental equipment

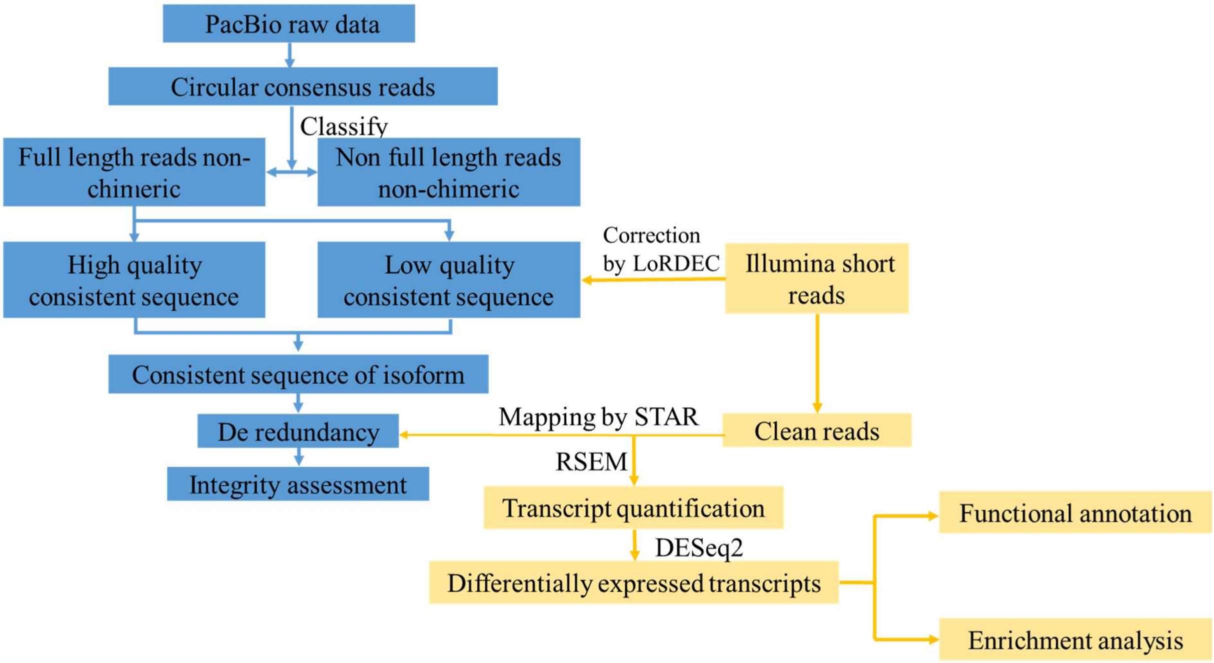

Figure S2 Relationship and key analysis procedures between PacBio sequencing and Illumina sequencing. The blue part represents the PacBio sequencing, and the yellow part represents the Illumina sequencing.

Table S1 Functional annotation of the key differential expression transcripts

Name	Functional description	Functional documentation
hsf1	heat shock transcription factor 1	*hsf1* mediates transactivation of heat shock genes and plays roles in dividing cells[1]
hsp90aa 1.2	heat shock protein 90, alpha, class a member 1, tandem duplicate 2	*hsp90ab1* (*hsp90* α family class a member 1) is associated with reproductive processes and cell proliferation[2, 3]
hsp90ab 1	heat shock protein 90, alpha, class b member 1	*hsp90ab1* (*hsp90* α family class b member 1) is associated with reproductive processes and cell proliferation[2, 3]
hspa9	heat shock protein 9	-
ldlrap1a	low-density lipoprotein receptor adaptor protein 1a	*ldlrap1a* is a ligand-binding site of the vitellogenin receptor[4]
lrp13	low-density lipoprotein receptor related protein 13	*lrp13* is a vitellogenin receptor in mediating yolk formation[5]
loc1005 36757	very low-density lipoprotein receptor-like	-
picalma	phosphatidylinositol binding clathrin assembly protein a	*picalm* bind to the cytoplasmic membrane to mediate yolk endocytosis[6]
arpc5lb	actin-related protein 2/3 complex subunit 5-like, b	*arpc5lb* (subunit of the *arp2/3* complex), which coordinates actin network formation, plays an important role in mediating clathrin endocytosis[7]
tuba1b	tubulin, alpha 1b	*tuba4l*, *tubb2b*, and *tuba1b* are the main constituents of tubulin[8, 9]
tuba4l	tubulin, alpha 4 like	*tuba4l*, *tubb2b*, and *tuba1b* are the main constituents of tubulin[8, 9]
tubb2b	tubulin, beta 2b	*tuba4l*, *tubb2b*, and *tuba1b* are the main constituents of tubulin[8, 9]
mapta	microtubule-associated protein tau a	-
kif15	kinesin family member 15	*kif15* plays a balancing role in transferring microtubule cytoskeleton into bipolar spindle[10]
kif20b	kinesin family member 20b	*kif20b* plays a positive role in driving and regulating cytokinesis during meiosis[11]
kif4	kinesin family member 4	*kif4* involves in chromosome formation, and drives microtubule sliding and chromosome compaction[12]
kif5c	kinesin family member 5c	*kif5c* main expressed in neurons and play a role in transport processes[13].
kifap3b	kinesin-associated protein 3b	-
dctn1a	dynactin 1a	*dctn1a* involves in microtubule binding to dynein, and acts as a key regulator for synthesizing dynein during microtubule transport[14]
dync1i2 a	dynein, cytoplasmic 1, intermediate chain 2a	*Dync1i2a* attaches to the spindle in the early stages of mitosis and relates to morphological mutation and apoptosis[15]
anln	actin binding protein	*anln*, as a highly conserved multi-domain protein, is associated with microtubules and plays an important role in cytokinesis[16, 17]
actb1	actin, beta 1	-
myh9b	myosin, heavy chain 9b, non-muscle	*myh9a* and *myh9b* are non-muscle myosin that present in every cell, which bind to the actin cytoskeleton to perform intracellular motor functions[18].
myh9a	myosin, heavy chain 9a, non-muscle	*myh9a* and *myh9b* are non-muscle myosin that present in every cell, which bind to the actin cytoskeleton to perform intracellular motor functions[18].
myo1b	myosin 1b	-
myo10l3	myosin x, like 3	-
mybph	myosin binding protein h	Myosin binding protein H (*mybph*) is considered to be related to autophagy[19].
mylk5	myosin, light chain kinase 5	-

Reference

1. Vihervaara, A. and Sistonen, L. HSF1 at a glance. *J. Cell. Sci.* **2014**, 127(2), 261-266. [CrossRef]
2. Dhamad, A. E., Zhou, Z., Zhou, J., Du, Y. and Didier, P. Systematic proteomic Identification of the heat shock proteins (Hsp) that Interact with Estrogen receptor alpha (ERα) and biochemical characterization of the ERα-Hsp70 Interaction. *Plos One*. **2016**, 11(8), e0160312. [CrossRef]
3. Seibert, J. T., Adur, M. K., Schultz, R. B., Thomas, P. Q. and Ross, J. W. Differentiating between the effects of heat stress and lipopolysaccharide on the porcine ovarian heat shock protein response. *J. Anim. Sci.* **2019**, 97(12). [CrossRef]
4. Shu, Y. H., Wang, J. W., Lu, K., Zhou, J. L., Zhou, Q. and Zhang, G. R. The first vitellogenin receptor from a Lepidopteran insect: molecular characterization, expression patterns and RNA interference analysis. *Insect Mol. Biol.* **2010**. [CrossRef]
5. Reading, B. J., Hiramatsu, N., Schilling, J., Molloy, K. T., Glassbrook, N., Mizuta, H., Luo, W., Baltzegar, D. A., Williams, V. N., Todo, T., Hara, A. and Sullivan, C. V. Lrp13 is a novel vertebrate lipoprotein receptor that binds vitellogenins in teleostfishes. *J. Lipid. Res.* **2014**, 55: 2287-2295. [CrossRef]
6. Scotland, P. B., Heath, J. L., Conway, A. E., Porter, N. B., Armstrong, M. B., Walker, J. A., Kl Eb Ig, M. L., Lavau, C. P., Wechsler, D. S. and Arun, R. The PICALM protein plays a key role in iron homeostasis and cell proliferation. *Plos One*. **2012**, 7(8), e44252. [CrossRef]
7. Papalazarou, V. and Machesky, L. M. The cell pushes back: The Arp2/3 complex is a key orchestrator of cellular responses to environmental forces. *Curr. Opin. Cell. Biol*. **2021**, 68: 37-44. [CrossRef]
8. Roll-Mecak, A. The tubulin code in microtubule dynamics and information encoding. *Dev Cell*. **2020**, 54(1), 7-20. [CrossRef]
9. Xu, Q. Q., Qin, L. T., Liang, S. W., Chen, P. and Chen, J. B. The expression and potential role of tubulin alpha 1b in Wilms' tumor. *BioMed Res. Int.* 2020, 2020:1-10. [CrossRef]
10. Reinemann, D. N., Sturgill, E. G., Das, D. K., Degen, M. S., V?R?S, Z., Hwang, W., Ohi, R. and Lang, M. J. Collective force regulation in anti-parallel microtubule gliding by dimeric Kif15 kinesin motors. *Curr. Biol.* **2017**. [CrossRef]
11. Li, G., Xie, Z. K., Zhu, D. S., Guo, T., Cai, Q. L. and Wang, Y. KIF20B promotes the progression of clear cell renal cell carcinoma by stimulating cell proliferation. *J. Cell Physiol*. 2019. [CrossRef]
12. Cross, R. A. and Mcainsh, A. Prime movers: the mechanochemistry of mitotic kinesins. *Nat. Rev. Mol cell biol.* **2014**. [CrossRef]
13. Campbell, P. D. and Marlow, F. L. Temporal and tissue specific gene expression patterns of the zebrafish kinesin-1 heavy chain family, kif5s, during development. *Gene. Expression Patterns Gep*. **2013**, 13(7), 271-279. [CrossRef]
14. Yong, D. L., Kim, B., Jung, S., Kim, H. and Kim, H. The dynactin subunit DCTN1 controls osteoclastogenesis via the Cdc42/PAK2 pathway. *Exper. Mol. Med*. **2020**, 52(3). [CrossRef]
15. Ansar, M., Ullah, F., Paracha, S. A., Adams, D. J. and Antonarakis, S. E. Article bi-allelic variants in DYNC1I2 cause syndromic microcephaly with intellectual disability, cerebral malformations, and dysmorphic facial features. *Am. J. Hum. Gen*. 2019, 104(6). [CrossRef]
16. Piekny, A. J. and Maddox, A. S. The myriad roles of Anillin during cytokinesis. *Semin. Cell Dev. Biol*. **2010**, 21(9), 881-891. [CrossRef]
17. Zhou, W., Wang, Z., Shen, N., Pi, W., Jiang, W., Huang, J., Hu, Y., Li, X. and Sun, L. Knockdown of ANLN by lentivirus inhibits cell growth and migration in human breast cancer. *Mol. Cell Biochem*. **2015**, 398(1-2), 11-19. [CrossRef]
18. Cheng, W., Zhou, X., Zhu, L., Shi, S. and Lv, J. Polymorphisms in the nonmuscle myosin heavy chain 9 gene (MYH9) are associated with the progression of IgA nephropathy in Chinese. *Nephrol. Dial. Transpl.* **2011**, 26(8), 2544-2549. [CrossRef]
19. Mouton, J., Loos, B., Moolman-Smook, J. C. and Kinnear, C. J. Ascribing novel functions to the sarcomeric protein, myosin binding protein H (MyBPH) in cardiac sarcomere contraction. *Exp. Cell Res*. **2015**, 331(2), 338-351. [CrossRef]
