# Supplementary figures and images for: Transcriptomic Analysis on the Effects of Altered Water Temperature Regime on the Fish Ovarian Development of Coreius guichenoti under the Impact of River Damming
Source: Biology (Basel). 2022 Dec 15;11(12):1829. doi: 10.3390/biology11121829 (PMC9775624; doi:10.3390/biology11121829)

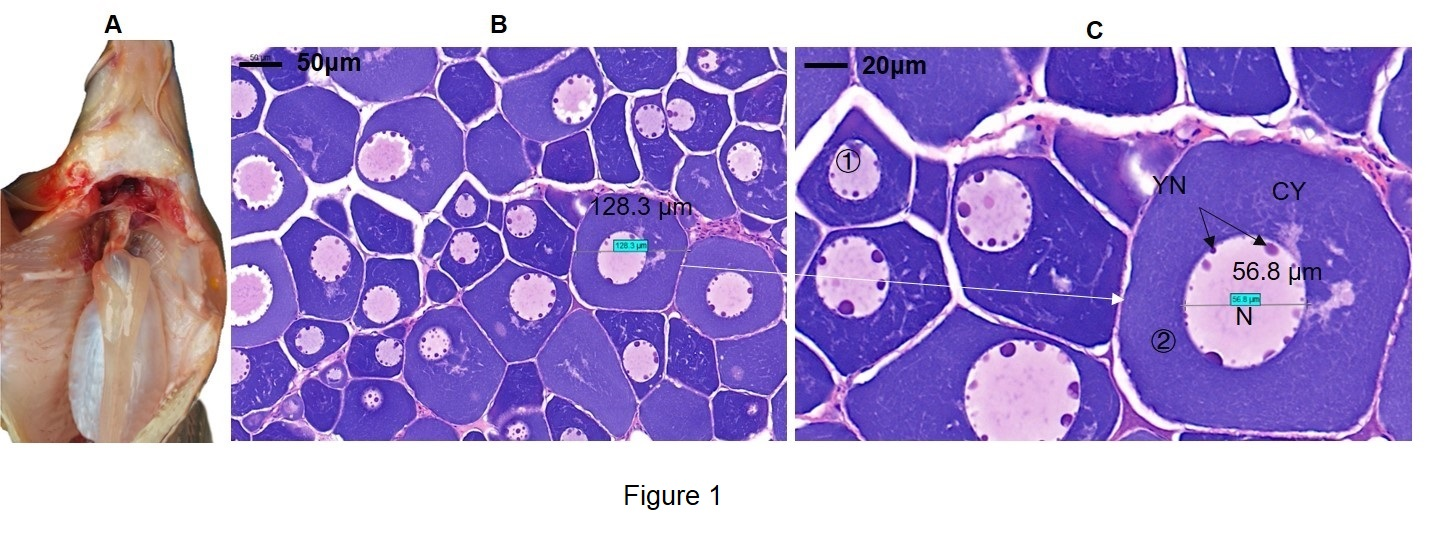

Supplement: Supplementary file 1 [file biology-11-01829-s001.zip › figure/1-f1.tif]

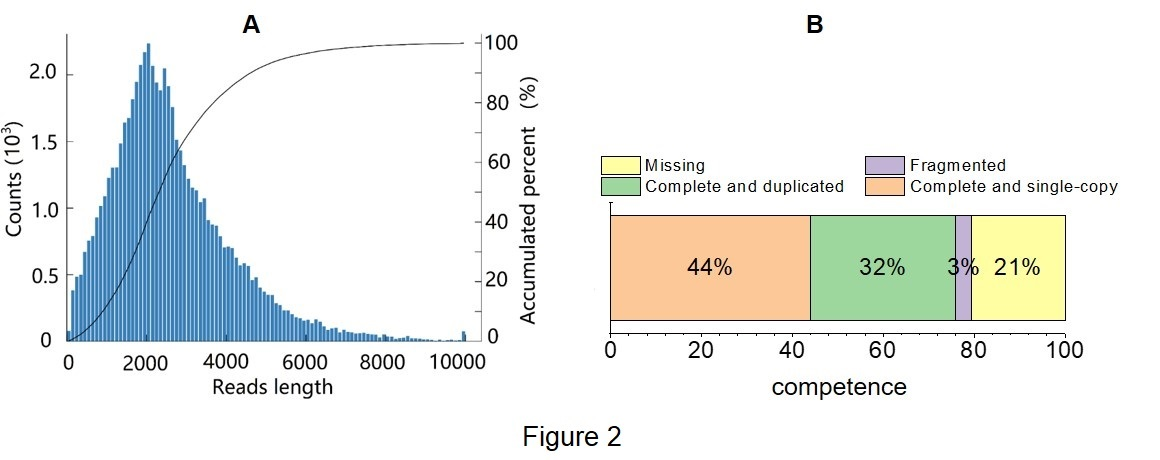

Supplement: Supplementary file 1 [file biology-11-01829-s001.zip › figure/2-f2.tif]

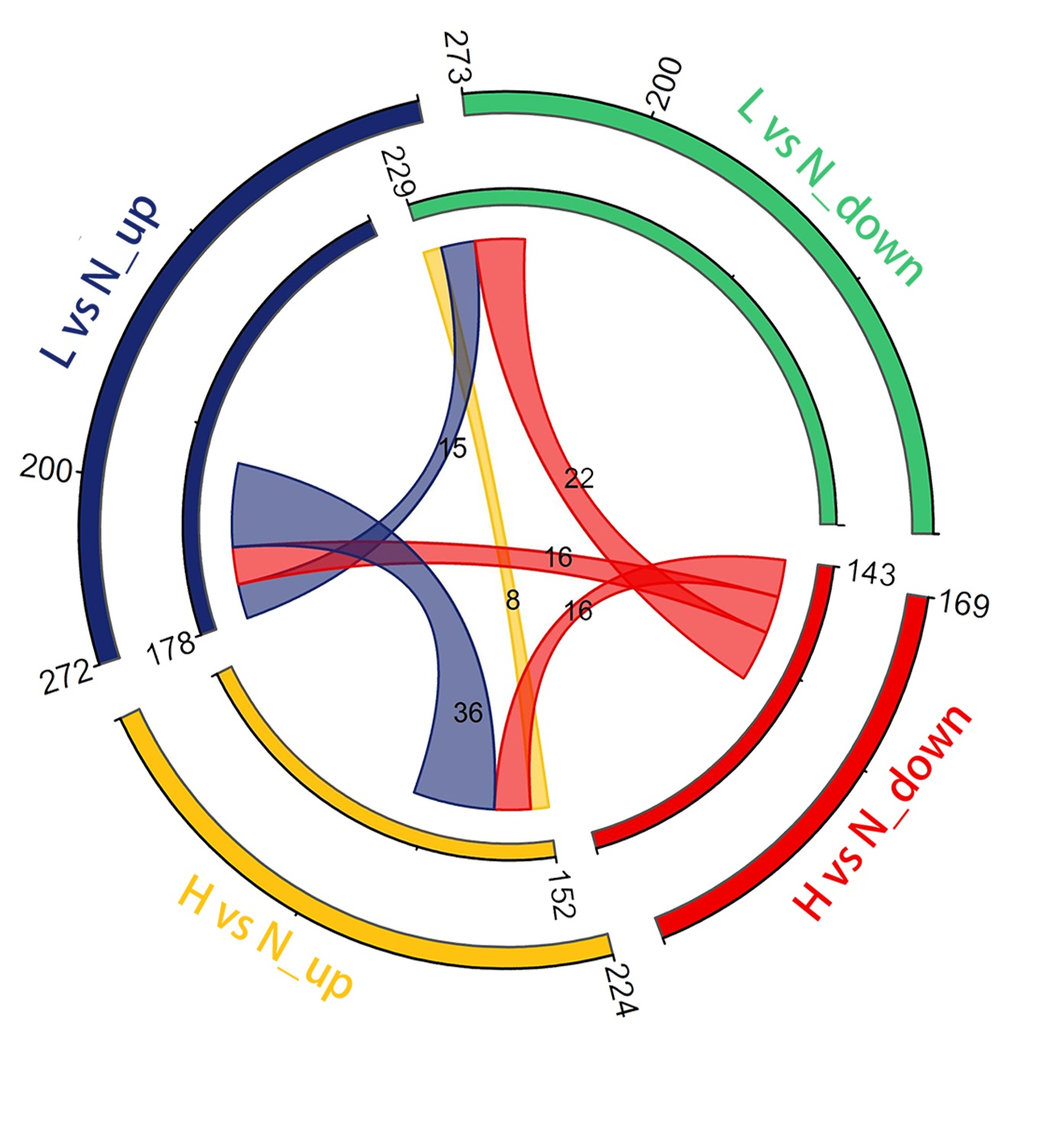

Supplement: Supplementary file 1 [file biology-11-01829-s001.zip › figure/3-f3.tif]

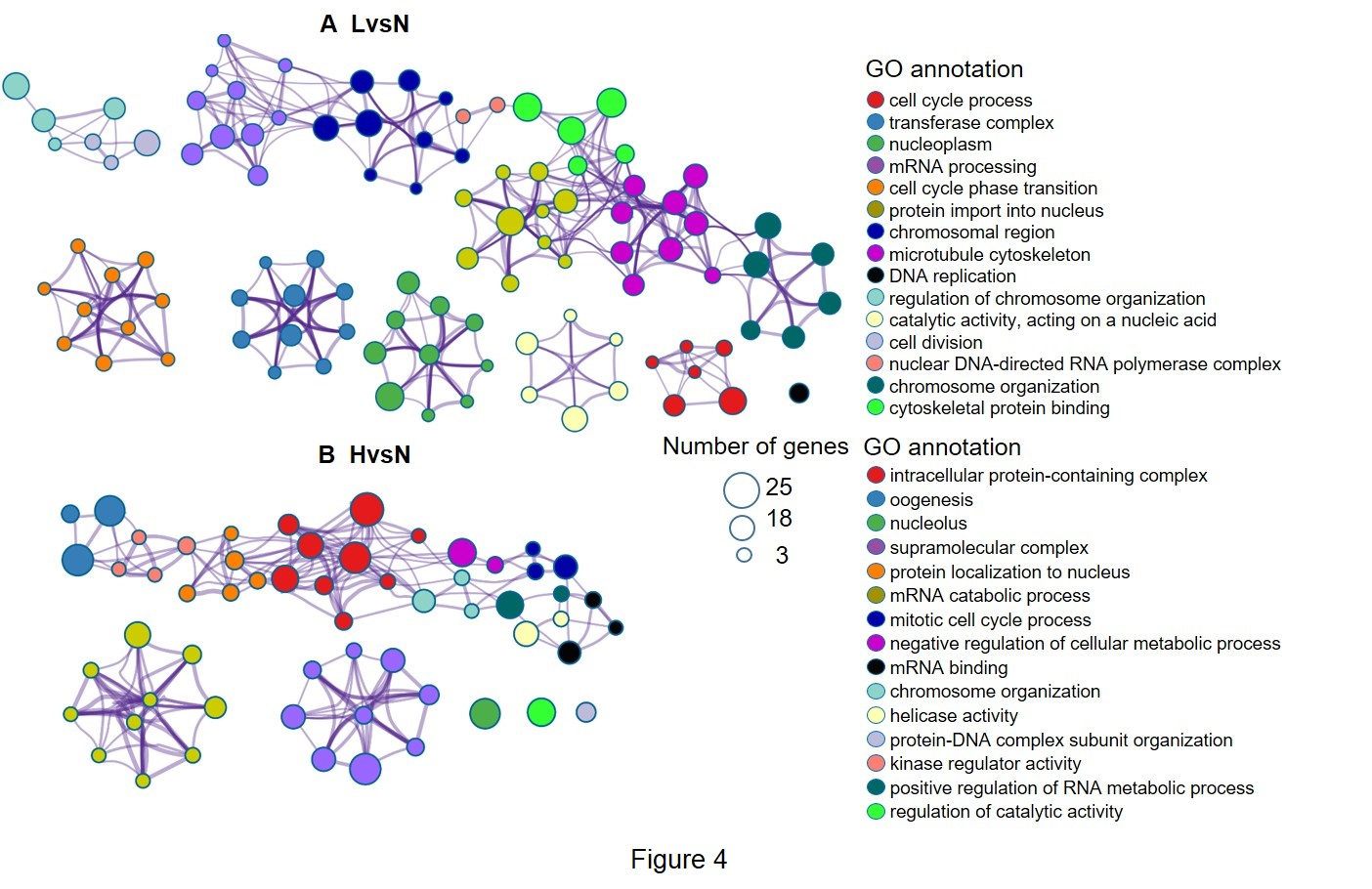

Supplement: Supplementary file 1 [file biology-11-01829-s001.zip › figure/4-f4.tif]

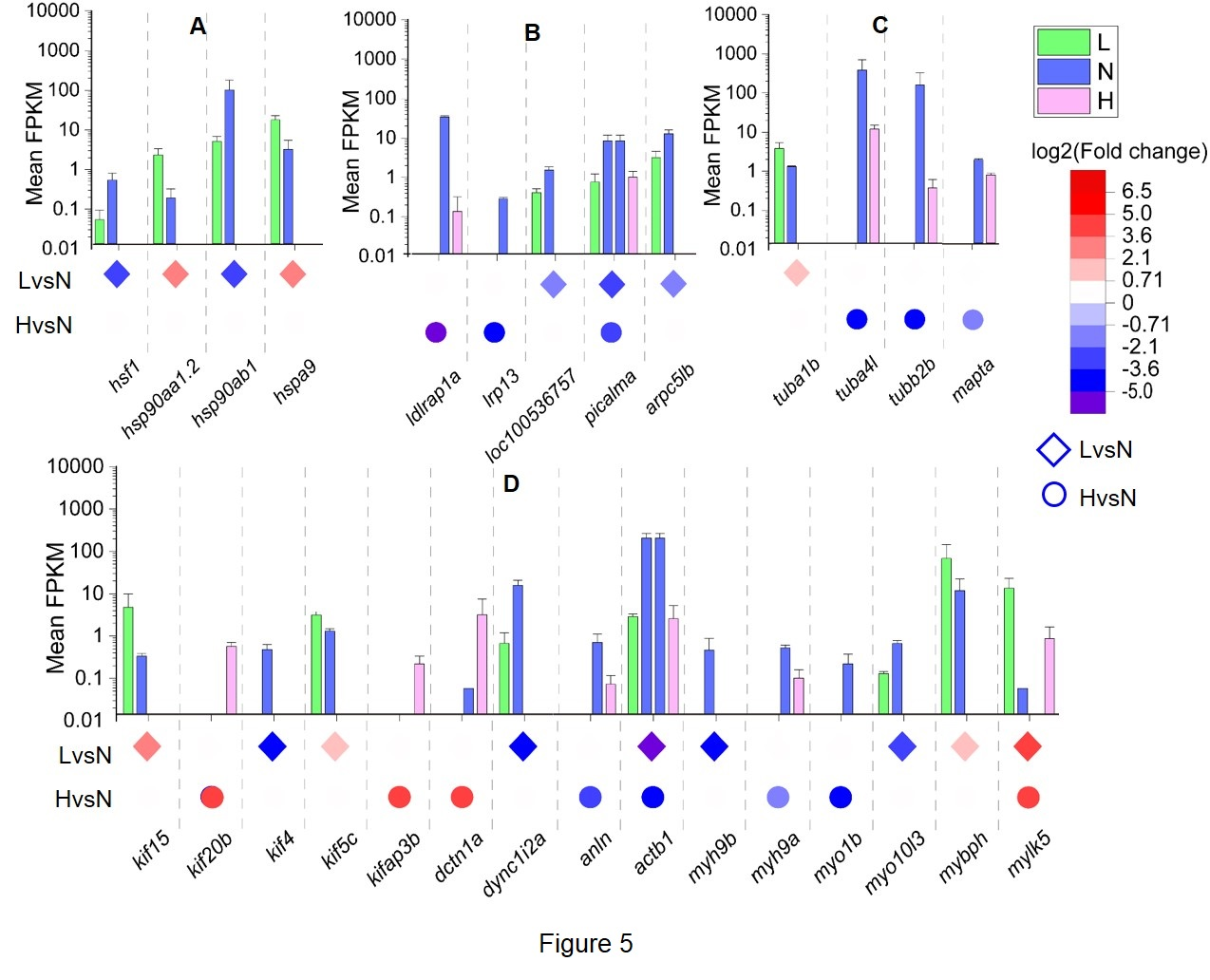

Supplement: Supplementary file 1 [file biology-11-01829-s001.zip › figure/5-F5.tif]

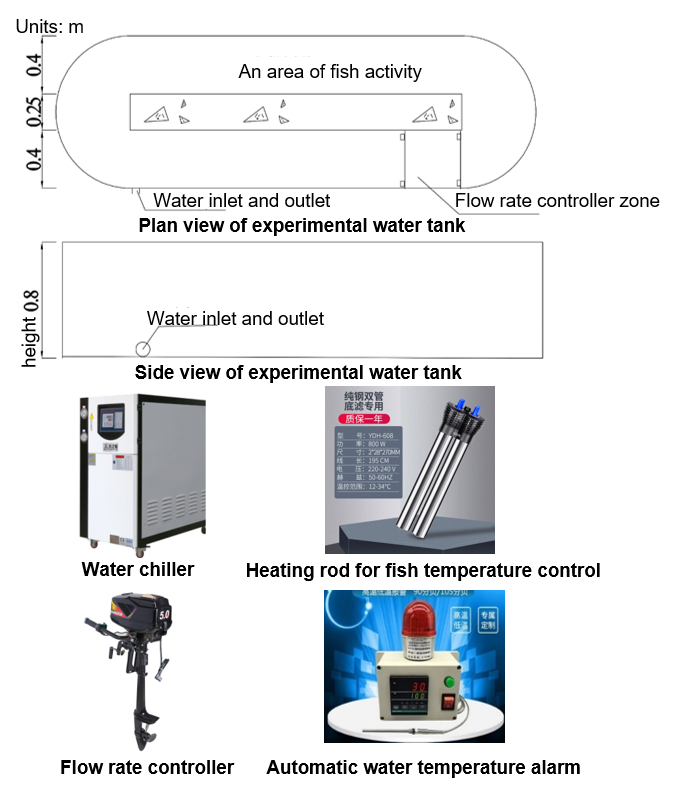

Supplement: Supplementary file 1 [file biology-11-01829-s001.zip › figure/Figure S1.png]

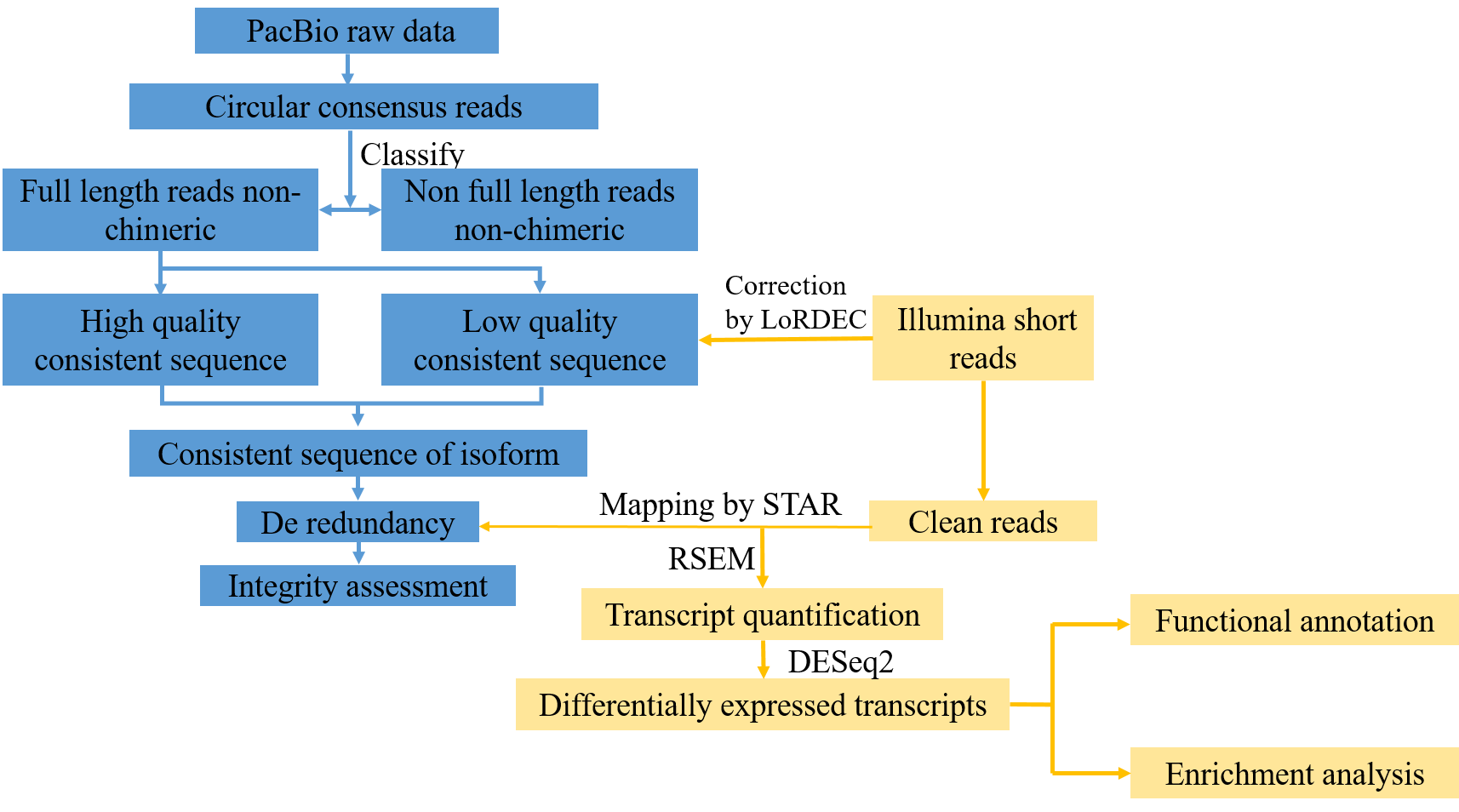

Supplement: Supplementary file 1 [file biology-11-01829-s001.zip › figure/Figure S2.png]

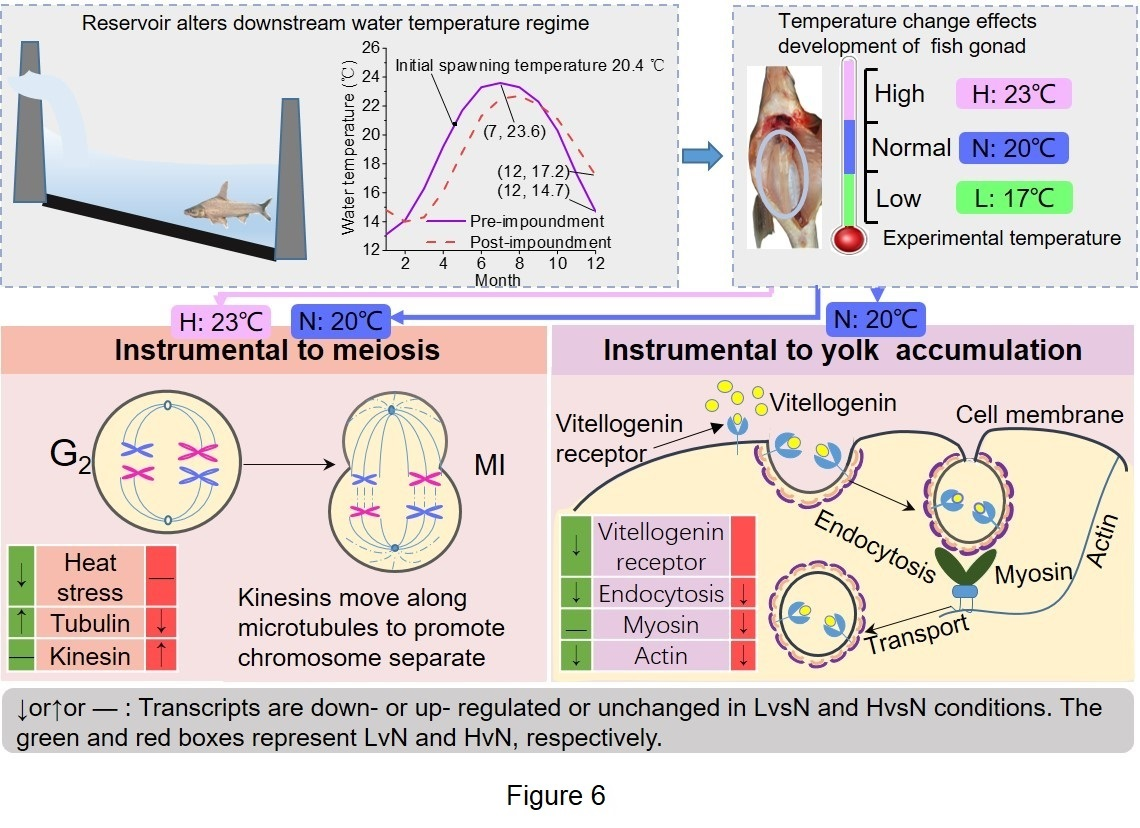

Supplement: Supplementary file 1 [file biology-11-01829-s001.zip › figure/graph abstract.tif]
